# Whole-Genome Sequence of Pseudomonas sp. Strain 1239, Isolated from Soil in Western France

**DOI:** 10.1128/MRA.01097-18

**Published:** 2018-09-13

**Authors:** Julien Crovadore, Damien Grizard, Romain Chablais, Bastien Cochard, Philippe Blanc, François Lefort

**Affiliations:** a Plants and Pathogens Group, Research Institute Land Nature and Environment, hepia, HES-SO University of Applied Sciences and Arts Western Switzerland, Geneva, Switzerland; b Proxis Développement, Levallois-Perret, France; c Bioprox, Noyant, France; University of Maryland School of Medicine

## Abstract

We report here the draft genome sequence of Pseudomonas sp. strain 1239, a bacterium that is potentially usable as a biostimulant for agriculture or in depollution. Its genome encodes resistance to mercury, heavy metals, and several antibiotics. It is potentially able to produce marinocine, a broad-spectrum antibiotic.

## Abstract (precis)

We report here the draft genome sequence of Pseudomonas sp. strain 1239, a bacterium that is potentially usable as a biostimulant for agriculture or in depollution.

## ANNOUNCEMENT

Pseudomonas spp. are aerobic rod-shaped and motile bacteria and ubiquitous residents of various terrestrial and aquatic environments (1). Very few are opportunistic pathogens of plants and animals. While most species are commensals, some are beneficial to plants (2–4) or usable in depollution (5, 6). Pseudomonas sp. strain 1239 was isolated from soil samples from the lower Loire Valley in western France and initially identified by biochemical profiling and morphology as Pseudomonas fluorescens, whereas 16S rRNA gene sequencing showed 99% shared identity with Pseudomonas putida.

DNA was extracted with a modified cetyltrimethylammonium bromide (CTAB) protocol (7) from a culture grown exponentially from a single colony in King B broth. A sequencing library was built with the TruSeq Nano DNA library preparation kit (Illumina, USA). Whole-genome sequencing (WGS) was performed using a MiniSeq high-output kit, within one Illumina MiniSeq run at 2 × 151-bp paired-end read length, and resulted in 309× genome coverage. The overall quality metrics of the reads were assessed with FastQC version 0.11.5 (8). Genome assembly was computed with the SPAdes genome assembler 3.10 (9), with a setting of “paired-end assembly, careful mode,” yielded 68 contigs (≥ 200 bp), was ordered with BioEdit version 7.0.5 (10), and was analyzed with QUAST version 4.6.3 (11), with the setting of “QUAST: skip contigs shorter than 200 bp.” The genome's total length is 6,024,399 bp, with a GC content of 64.03% and an *N*_50_ value of 244,210 bp.

A blast analysis of the complete 16S rRNA gene showed that this strain shares 82% identity with Pseudomonas alkylphenolica sp. nov. strain KL28 (12). Automated gene annotation carried out by the Prokaryotic Genome Annotation Pipeline (PGAP) version 4.1 (13) identified 5,538 coding sequences and 89 RNA genes, while RAST version 2.0 (14), using the ClassicRAST annotation scheme, detected 5,376 coding sequences and 76 RNA genes. RAST found a partial prophage genome (24 genes) on contig 61. PlasmidFinder version 1.3 (15) and plasmidSPAdes (16), both using default settings, did not detect any plasmids. Five genes code for auxin synthesis. Siderophore sensing, transport, and reception are encoded by 34 genes, while 26 other genes encode siderophore synthesis and secretion, with 19 genes arranged in a pyoverdin gene cluster. This strain also has two complete type II and VI secretion systems (T2SS and T6SS, respectively) organized in operons. Two genes, *lodA* and *lodB*, might encode the production of marinocine, a broad-spectrum antibiotic (17, 18). Degradation of aromatic compounds is suggested by 118 genes. Like Pseudomonas putida strain DRA525 (19), the presence of a mercury ion reductase gene and some genes of the mercury operon (*merA, merP, merT,* and *merR*) would ensure resistance to mercury. Similarly, multiple copies of the genes *arsB, arsC, arsH, arsR,* and *acr3* would provide resistance to arsenic. A putative resistance to heavy metals is provided by 73 genes, including multiple copies of the genes *czcD, czcA, czcC, cusB* or *czsB* *, cusA,* and *czrR* and genes coding for heavy metal sensor histidine kinases. A complete MexE-MexF-OprN multidrug efflux system predicts tolerance to heavy metals and antibiotics. These characteristics make this strain interesting for agriculture and soil depollution.

### Data availability.

This whole-genome shotgun project was deposited at DDBJ/EMBL/GenBank under the accession number NFSA00000000. The version described in this paper is the first version, NFSA01000000. The 68 contigs have been deposited under the accession numbers NFSA01000001 to NFSA01000068. Raw sequencing data sets have been registered in the NCBI Sequence Read Archive database (20) under the accession number SRR5515064.
